# Cognitive, social, and mental health functions of refugee children – screening and supportive actions at school: a study protocol

**DOI:** 10.1186/s40359-024-01752-3

**Published:** 2024-05-07

**Authors:** Oskari Lahtinen, Samuli Kangaslampi, Sanni Aalto, Joosu Soosalu, Kirsi Peltonen

**Affiliations:** 1 INVEST Research Flagship Centre, University of Turku, Turku, FI-20014 Finland; 2 Faculty of Social Sciences / Psychology, Tampere University, Tampere, Finland

**Keywords:** Refugee, Children, School, Cognitive functioning, Mental health, Trauma

## Abstract

**Background:**

Despite a world-leading educational system, an achievement gap in educational outcomes exists between children of refugee background and native-born peers in Finland. To offer targeted support for children at schools, we need to be able to reliably assess and understand the interplay of the aspects of children’s cognitive, social, and mental health functions that may explain the underachievement of refugee children.

This study tests a novel research-based, universally applicable screening battery for evaluating cognitive, social, and mental health functioning of children at schools and planning supportive actions. It aims to answer research questions about a) the cognitive, social, and mental health functioning of refugee children compared with non-refugee immigrant and native-born children, b) the interplay of these different functions among refugee and other children, c) whether implementing a screening battery can inform schools in planning supportive actions for (refugee) children, and d) whether such supportive actions result in improvements in cognitive, social, and mental health functioning.

**Methods:**

Four hundred fifty children aged 10–12 will be recruited from primary schools, including 150 children of refugee background, 150 of non-refugee immigrant background, and 150 native-born Finnish children. A screening battery including tasks and questionnaires on different aspects of cognitive, social, and mental health functioning will be used to assess the children in their classrooms at the start and end of a school year. Supporting information will also be collected from parents and teachers. The information gathered will be collated into class-level feedback reports for teachers and, with parental permission, individualized reports for multiprofessional student welfare bodies, for informing supportive actions. Correlational and latent profile analyses, ANOVAs, and linear regression will be used to answer the research questions.

**Discussion:**

This study will help clarify how the interplay of cognitive, social, and mental health factors may explain underachievement at school among refugee children. It will provide evidence about the extent to which a standardized screening battery could be helpful in informing and planning supportive actions for children at schools, and whether such supportive actions can lead to positive cognitive, social, or mental health outcomes.

**Trial registration:**

The study will be preregistered on the Open Science Framework.

## Background

### Aims and objectives

As refugee children from all over the world enter European classrooms, policy makers, educators, and researchers need to rethink their education “at home” in order to ensure quality and equality. At the end of 2022, 108.4 million people were forced to flee their homes due to conflicts, violence, fear of persecution and human rights violations, with more than half of this population children and adolescents [1]. In Europe, the war in Ukraine alone has forced millions of people to leave their homes. Around 12 000 Ukrainian children had arrived in Finland by the fall of 2022 [2]. Learning opportunities are vital for immigrant children’s wellbeing and life satisfaction [3]. Unfortunately, both Finnish and international comparative studies show that migrant children commonly underachieve academically, meaning that their school performance does not correspond with their cognitive capacity [4–6]. Even if Finland has a world leading educational system [7], these challenges are pronounced among refugee students. A recent comparative analysis of educational outcomes among refugee children in the Nordic countries shows that refugee students have lower school results than their native-born peers in compulsory education [8]. In Finland, the achievement gap in mathematics has been found even greater than in other Nordic countries, corresponding to two years of studies [9].

During our previous work with Finnish schools [10], we learned that a major reason for poor school performance among refugee and non-refugee immigrant children lies in difficulties detecting the core reasons and mechanisms behind learning barriers. The reasons and mechanisms involve cognitive, social, and mental health issues that should be assessed early on and in combination with each other. Further, Nordic schools have varying, and often insufficient, knowledge and competence on how to relate appropriately to a diverse group of refugee students with multifaceted needs [8]. In Finland, adolescents with immigrant backgrounds are at greater risk of unmet needs for support and help at school than native-born Finnish adolescents [11] and teachers feel that immigrant students lack consistent support structures [12]. We need objective, universal, and user-friendly tools to assess different factors that explain children’s capacity to follow teaching or carry out their tasks at school, which are at the core of children’s integration into the education system. Profile-based scientific information about relative, age-specific differences across refugee, non-refugee immigrant, and native-born children regarding cognitive and social capacity and mental health is not available, and supportive actions are therefore often ineffective.

One reason for migrant children’s academic underachievement may be their stressful experiences. Coping with these experiences uses up resources that would otherwise be used for learning. Pre- and postmigration traumatic experiences have indeed been found to be major risk factors for learning problems [13]. Stressful events are associated with mental health symptoms in a dose-dependent manner [14, 15] and as many as 20–48% of refugee children suffer from of posttraumatic stress disorder (PTSD) and depression, although many also show resilience [16, 17]. A recent nationwide, population-based study (*n* = 73 690) showed that first generation immigrant adolescents in Finland had significantly more mental health symptoms than their second-generation immigrant peers [18]. Studies among adults have repeatedly shown that struggling with trauma symptoms such as re-experiencing and avoidance may impair cognitive functioning, including general intelligence, executive function, attention, verbal skills, memory, and visuospatial skills (e.g., [19, 20]). Some researchers have also detected effects of such experiences on children’s abstract reasoning, concentration, memory and attention [21–23]. Moreover, diminished ability to think, concentrate, and make decisions are core symptoms of depression [24].

All these functions form the basis for learning at school, but research is lacking among children and adolescents. The vicious circle of trauma exposure also presents itself in findings that more trauma exposure may jeopardize social competence [25] and that students who have poor social relations also have more problems in academic adjustment [26, 27]. In addition, trauma survivors who have problematic peer relations exhibit more mental health problems [28–30]. There is some evidence that level of social competence may be associated with aspects of cognitive functioning mentioned above, but the studies so far have been carried out among adults [31] and very young children [32]. Again, we need full elaboration and replication with school-aged children.

In sum, we need to understand the interplay of children’s cognitive, social, and mental health functions to offer targeted and tailored help for children at schools. This is important for all children, but would also benefit immigrant and especially refugee children, for whom uncovering the challenges underlying school performance has for a long time been seen as challenging. Our project develops a research based, validated and universal tool, the Screening Battery (SB), for evaluating and supporting further action toward better cognitive, social, and mental health functions. In this project, we develop, pilot and implement this tool in Finnish schools, and collect groundbreaking data on the interplay of cognitive, emotional, and social mechanisms that may underlie school problems among refugee children. We will compare profiles between refugee children and non-refugee immigrant children as well as their native peers in these dimensions. We will also study the effectiveness of supportive actions informed by the results of the screening battery, and planned by the school, while controlling for other significant life changes over the study year.

### Research question

First, we want to find out how the cognitive, social, and mental health functions of refugee children compare with (a) same aged immigrant children without refugee background and (b) native-born children in Finland. We will also examine whether worse mental health (trauma-related and depressive symptoms) is linked with poorer cognitive function and social competence across the sample. We hypothesize based on earlier (limited) literature in the field, 1) that refugee children, for whom exposure to trauma is likely more common, exhibit lower levels of cognitive functioning compared to immigrant children without refugee background and native Finnish children, and 2) that mental health problems and poorer social competence explain this link.

Second, we will examine whether reliable evaluation of cognitive, social, and mental health functions of students can inform schools about supportive actions for refugee children and whether this process is different compared with non-refugee immigrant children and/or native-born Finnish children. We will look at whether children who scored above/under critical thresholds on our Screening Battery (SB) report receive supportive actions during the school year. If they do, we look at what kinds of actions inside and outside the classroom schools conduct for refugee children, and whether the output of the SB was utilized to plan this support We will further look at whether the actions are different compared to actions with non-refugee immigrant children and/or native-born Finnish children and whether the actions are different compared to actions that do not use the information produced by the screening battery.

We will also examine whether the supportive actions are reflected in cognitive, social, and mental health functions of refugee children within their first year in Finnish schools (after controlling for other significant life changes during the study year). We will investigate whether this effect is different compared to non-refugee immigrant children and/or native-born Finnish children and if the effect is different compared to actions not using the information produced by the screening battery. We hypothesize 3) that supportive actions informed by the findings of the screening battery have a stronger positive effect than other actions. Finally, we will examine whether improvements in mental health (trauma, depression, resilience) and social functioning during the school year are associated with better performance in cognitive tests.

## Methods

### Participants and procedure

The participants are a sample of 450 students aged 10–12 from primary schools in Southwest Finland. 150 children are refugees, 150 non-refugee immigrants, and 150 native-born Finnish schoolchildren. Schools are selected on the basis that they have a preparatory class where all asylum seekers and most non-refugee immigrants study for their first year after arriving in Finland. We will select one preparatory class and one regular class in each school to draft the sample for the study. The study has two time points: baseline measurements at the beginning of the school year (T_1_) and follow-up measurements at the end of the school year (T_2_). The study uses a screening battery tool to administer a variety of questionnaires and tests which are then used to give feedback about the students to teachers and multiprofessional student welfare bodies. We aim to collect all the data within one school year (2024–2025), but if the number of participants does not reach 150 in each group, a second round of data collection will take place in 2025–2026. We will conduct a small-scale pilot study in one preparatory and one regular class in Spring 2024.

### Screening battery

The screening battery (SB) gathers many kinds of data about the participants using demographic survey questions, tasks, and questionnaires. The children answer questions and complete tasks, and data is also collected from parents and teachers. The battery contains standardized and universally applicable assessments of a) cognitive functions (general intelligence, working memory, self-regulation, and self-efficacy and decision making), b) mental health (symptoms and well-being), and c) social competence. Prior to T_1_, the research group will offer a half-day training to teachers and school psychologists participating in the study on how to use the screening battery and specifically its feedback function. During the training, initial ideas and resources on how to transform the feedback into supportive actions at schools will be discussed among participating teachers and school psychologists.

The screening battery uses the Gorilla online platform to administer questionnaires and tasks (https://gorilla.sc/). Results are then transferred via an API to a Microsoft Power BI feedback tool which compiles both anonymous general (for teachers) and personalized (for multiprofessional student welfare bodies with consent from children’s parents) feedback with data visualizations and recommendations for supportive measures for students. At the end of the school year, the SB is administered again for the same classes (T_2_). The data about any supportive actions (either using the information produced by SB or not) that children may have received during the school year is also collected. The data is collected from parents, teachers, and multiprofessional student welfare bodies. The child’s own experiences of the support received are also collected during follow-up measurements. The researchers, who are all licensed psychologists and specialists in child cognitive and social skills as well as in child mental health, will be available for consultations with the schools throughout the study year, in case the school has further questions or concerns.

### Feedback tool

The feedback tool collates information on participants’ cognitive, social, and mental health functioning and compiles two reports: one for the teacher and one for school health staff. The teacher report is at a classroom level and presents results as anonymized averages. The teacher data includes a categorical graphical representation (“low”, “average”, “high”) of self-regulation, intelligence, self-efficacy, resilience, depression, trauma, loneliness, altruism, and language development levels in the classroom, with clarifying depictions of these qualities and guidance for their interpretation written by clinical psychologists working in the research project. The school health service report involves individual student data. Upon request, and with the permission of the parents, the multi-professional student welfare body is offered detailed data on the individual’s results on the screening battery. This includes a graphical summary of cognitive, social, and mental health data gathered in the study compared to subsample means (refugee, immigrant, native Finnish) for each child on each variable. The individual results can also be discussed with the child him/herself.

### Tests and measures

**Demographic variables.** At T_1_, we ask the children, as well as their parents, about the children’s age, class, gender, living arrangements, and country of birth. If the country of birth is not Finland, we ask how many years they have spent in Finland, with whom they came to Finland, immigration status, place of residence, and reason for migration. At T_2_, we ask about significant life changes after T_1_, including changes in legal status in host country, family composition, significant losses or other changes in close relations, and other significant life changes. The same kind of questionnaire was used in a previous study among refugee and native-born children in Finland [10, 33]. At T_2_, we collect a list of supportive actions the child thinks they have received during the school year.

#### Cognitive functioning

**General intelligence** is measured with a matrix reasoning task. This task involves 15 trials, where participants are presented with a 3 × 3 matrix with a missing piece and four options for the missing piece to pick from to best complete the matrix. The 15 matrix items, ranging from easy to quite difficult, were selected from the Matrix Reasoning Item Bank [34] to provide adequate differentiation. The total number of correct choices is summed, with participants’ performance compared to each other, as no norms for this selection of items exist.

**Working memory** facilitates learning, comprehension and problem-solving as it allows an individual to keep information in mind and use it to execute cognitive tasks. Working memory is assessed by a digit span task, where the participant is shown numbers one at a time in sequences of increasing length. The participant then tries to repeat the sequence by clicking numbers on the screen.

**Self-regulation** is the ability of an individual to flexibly apply situational rules and adjust his/her actions and expectations. To succeed, individuals must follow legal and cultural rules, regulate impulses, and apply different rules depending on contexts. Self-regulation is measured with one task per each of the three dimensions of self-regulation: a) Inhibition, b) Shifting, and c) Updating. *Inhibition* is measured with the Go/NoGo task [35] involving the ability to prevent a response or impulse. Most trials are “Go” or “Button Press” trials, however on the rare “No Go” trials, individuals must inhibit a button press. *Shifting* is measured with the Colour Shape Task [36], involving shifting between tasks flexibly. Trials randomly alternate between classifying by shape or by color. *Updating* is measured with the Corsi Task [37], which requires adding and deleting information in working memory. In this task, boxes light up in sequences, and individuals must then correctly click them in the same sequence. Each level adds difficulty by adding another box to the next new sequence.

**Decision making** is assessed with the Balloon Analog Risk Task [38]. In this task, participants collect points by inflating balloons. Each time the participant presses a button, a balloon inflates and the points value of the balloon in question is increased. However, the balloon also has a chance of exploding at each inflation. If the balloon explodes, the participant loses all points for that balloon. Instead of inflation, the participant can also choose to bank current points and move on to the next balloon. Different colored balloons with different chances of exploding are presented, and the participant may learn to assess the level of risks.

**Self-efficacy.** Self-efficacy is an individuals’ belief that through perseverance, they can reach a goal. Self-efficacy is also commonly referred to as ”grit” or ”motivation”. Self-efficacy is measured using the 10-item academic self-efficacy subscale of the Self-Efficacy Questionnaire for Children [39]. The subscale measures an individual’s conception of his/her own abilities related to success at school. Participants assess on a five-point scale how well each question describes their behavior (1 = *not at all* to 5 = *very well*).

#### Social functioning

**Social competence** is the ability to understand others’ emotional states and perspectives and use this information appropriately to guide decision making. Social competence is highly valued and rewarded in society both in personal relationships as well as peer group integration. Social competence is measured with two tasks, one each for emotion reading and theory of mind.

**Emotion reading** is measured with the Reading the Eyes task [40]. Photographs of eyes are presented together with four options for different emotions, and the participant must select the one matching the expression depicted. The share of correctly identified emotions yields a score of mentalizing accuracy.

**Theory of mind** is measured with The Yoni Test [41, 42]. In this test, children are presented with a series of trials where a cartoon outline of a face is looking at different objects or people around it. They must select the correct object or person the character is thinking about or has feelings towards based on its gaze, facial expressions or the facial expression of other faces it is referring to. Half the 64 trials require first-order and half second-order inference.

**Prosocial and altruistic sharing** is measured by the experimental protocol implemented in [43], extended from the work of Fehr et al. [44]. Each participant makes four choices between two options each. Each option describes an allocation of x units of rewards to the decision maker and y units to an anonymous recipient (of the same gender and roughly the same age). In each of the four choices, one allocation (x, y) is always the allocation (1, 1), while the alternative allocation was designed to classify different social preference types. The types are defined as follows: (1) altruistic if subjects maximize the recipient’s payoff in all four choices; (2) egalitarian if they always minimize the difference in payoffs for themselves and the recipient, which means to choose always the allocation (1, 1); (3) spiteful if they always minimize the recipient’s payoffs; and (4) selfish if they maximize their own payoffs in the first and the fourth choice (the payoff of the decision maker is the same in both options of the other two choices).

**Loneliness in the peer context.** The 16-item Peer Network and Dyadic Loneliness Scale (PNDLS) [45] is used to assess the students’ experiences of loneliness in peer relations on two levels, with 8 items each: peer network loneliness (i.e., loneliness associated with peer group isolation) and peer dyadic loneliness (i.e., loneliness associated with the absence of a close, enduring, emotionally intimate friendship with a specific other peer). The student reads two descriptions of different types of children and answers which describe them better. The student then evaluates on a two-point scale whether that description is *sort of true* or *really true* for them.

#### Mental health

**Resilience** is measured with the Individual Resiliences Questionnaire developed and found reliable in a previous study [33], after considering the well-validated Child and Youth Resilience Measure (CYRM) [46], but finding some of its items difficult for the target group of refugee children. The questionnaire consists of 10 items measuring the positive individual resources of children and adolescents. Participants evaluate on a three-point scale how well each description fits them (0 = *not at all*, 1 = *somewhat*, 2 = *yes, fits well*).

**Traumatization.** The Child and Adolescent Trauma Screen (CATS) [47] will be used to measure trauma exposure and trauma symptoms. This scale first measures exposure to 15 different potentially traumatic events. If the child has experienced any of these traumatic events, he/she will answer 20 items measuring posttraumatic stress symptoms. This screening test is based on DSM-5 criteria for PTSD and includes symptoms of re-experiencing, avoidance, negative alterations in mood and cognitions, and hyperarousal. The child answers on a 4-point scale how much the described symptoms have bothered them during the last two weeks (0 = *never*, 1 = *once in a while*, 2 = *half the time*, 3 = *almost always*).

**Depression** is measured with the Birleson Depression Self-Rating Scale (DSRS) [48]. This 18-item scale is based on an operational definition of depressive disorder, and the child answers on a 3-point scale how often (0 = *Mostly*, 1 = *Sometimes*, or 2 = *Never*) the statements have applied to them during the past week. The scale has been widely used across diverse ethnic and national groups [49].

All mental health measures have good reliability among war-affected children and adolescents [47, 50, 51] and we have used most of them in our previous studies among refugee children [10, 33, 52, 53].

#### Parental report

**Demographic variables.** At T_1_, we ask background information such as parents’ age, country of birth, education, marital status, and housing arrangements are collected. We also ask parents to report on the child’s country of birth, current legal status in host country, time in host country, and reason of migration. We request this information from both parents and children. Parents would be more reliable informants on these questions, but in the likely situation that we are unable to reach all parents, we will have the child’s report on the same topics. If there is discrepancy in child and parent reports, we rely on parents’ report. At T_2_, we ask about significant life changes after T_1_. The questionnaire includes changes in legal status in host country, family composition, significant losses, or other changes in close relations and other significant life changes. At T_2_, we collect information on whether or not the information provided by the SB has been used for the child, and a list of supportive actions the child or family has received.

**Family functioning** is measured by 12 items from the General Functioning subscale of the McMaster Family Assessment Device (FAD) [54, 55]. The parent assesses on a four-point scale how much they agree with the descriptions of family functioning (0 = s*trongly agree*, 1 = a*gree*, 2 = d*isagree*, 3 = s*trongly disagree*).

**Child language and development** is measured by 10 selected items from the Alberta Language and Development Questionnaire (ALDeQ) [56]. The selected items were chosen to reflect the parents’ view of the general development of the child and possible challenges in it, as well as parents’ own reading or learning difficulties. The parent evaluates each item on a four-point scale, but due to the varying form of the items, the answer categories vary between items (such as 0 = *never*, 1 = *rarely*, 2 = *sometimes*, 3 = *very much* or 3 = *very easy*, 2 = *easy enough*, 1 = *sometimes not easy*, 0 = *no, very hard*).

#### Teacher report

**Teacher self-efficacy** in the classroom is measured by Teachers’ Sense of Efficacy Scale (TSES). The TSES is a 12-item questionnaire, where teachers indicate on a 9-point scale how much they can do as teachers in each of the situations described (1 = *nothing*, 3 = v*ery little*, 5 = s*ome influence*, 7 = *quite a bit*, 9 = *a great deal*).

**Teachers' view of children’s co-operation skills and classroom atmosphere.** This quality is measured with a 9-item questionnaire developed in the Yhteispeli project in Finland [57]. Teachers respond on a 5-point scale how well the statements fit him/her.

At T_2_, we collect information on whether or not the information provided by the SB has been used and a list of supportive actions in the classroom.

**Teachers’ beliefs and perspectives on teaching for social justice** are measured with nine items of the original 12-item Learning to Teach for Social Justice-Beliefs (LTSJ-B) scale [58], because the nine-item adaptation has previously been adapted and found suitable for the Finnish school context [59]. The teachers assess items related to culturally responsive education on a five-point scale (1 = s*trongly agree*, 2 = a*gree*, 3 = *I don’t know*, 4 = d*isagree*, 5 = s*trongly disagree*).

#### Analysis plan

**Power.** In order to form profiles across different groups of children and to assess changes in cognitive, social, and mental health functions across those children who will receive SB based supportive actions and those who do not, we performed a preliminary power analysis to determine the sample size required to detect medium-sized or larger effects on these primary outcomes. Based on typical sizes of Finnish preparatory classes, we estimated the average size of each cluster to be 17, with a variance of 10. Intraclass coefficients for these outcomes are challenging to estimate, but based on our previous cluster-randomized research among war-affected children, values of 0.01–0.1 could be reasonable. With a relatively conservative estimate of 0.07 for the ICC, and setting alpha at 0.05, to achieve 80% power to detect a medium-sized (d = 0.5) or larger effect, we would need about 150 children in each group, meaning approximately 450 children in total.

**Analyses.** Means, standard deviations, and correlations will be evaluated for all variables reported by children, parents, and teachers. To test Hypothesis 1, baseline differences between the study’s three groups will be investigated using 3-way ANOVAs and followed up with *t*-tests to compare means. To test Hypothesis 2, correlation analyses will first be used to examine whether mental health (trauma-related and depressive symptoms) is associated with poorer cognitive function and social competence. Latent profile analyses (using the TidyLPA package in R) will then be run for the entire sample under each of the three dimensions: cognitive, social, and mental health. Emerging profiles will be compared with how they line up with our three groups (refugee, immigrant, native-born Finnish).

To test Hypothesis 3, screening battery effects on cognitive, social, and mental health functioning will be evaluated using repeated measures ANOVAs with 2 × 3 for {T_1_, T_2_} and {children who received SB-based support, children who received non-SB-based support, children who did not receive SB-based support} among children who scored above/under critical thresholds, and following up with *t*-tests to compare means. Differences in SB-based actions will also be evaluated qualitatively. We will further use linear regression to test whether changes in mental health (trauma- and depressive symptoms) and social functioning associate with changes in cognitive functioning from T_1_ to T_2_. In addition to analyses in the whole sample, we will also do these analyses by subgroup, separately for our three groups. Classroom atmosphere, teacher self-efficacy and awareness of culturally responsive education, family functioning, child language and development as well as children’s cultural background will be used as covariates in the regression analyses and ANOVAs. Effect sizes and confidence intervals will be reported for main effects and p-value based inference will be de-emphasized based on guidelines for New Statistics [60].

## Discussion

To be effective in targeting academic underachievement, supportive actions at schools should be based on both scientific evidence about what lies behind such underachievement in general and on specific personalized information about what the student in question needs. This study aims to provide evidence and increase our understanding of both factors. More specifically, it will provide information on the extent to which a standardized screening battery gathering information on cognitive, social, and mental health factors from multiple sources can inform and help plan supportive actions, and whether supportive actions based on the findings of such a battery in turn lead to positive cognitive, social, or mental health outcomes.

The findings and the developed screening battery, intended to stay in use after the research project finishes and spread more widely, may be useful for all children but could be especially valuable for children of immigrant and refugee background, among whom the precise factors underlying academic underachievement remain unclear.

## Data Availability

No datasets were generated or analysed during the current study.
